# Application of Gastroscopy in the Diagnosis of Congenital Pyriform Sinus Fistula in Children

**DOI:** 10.3389/fped.2020.541249

**Published:** 2021-01-25

**Authors:** Shengcai Wang, Lin Mei, Yanzhen Li, Xuexi Zhang, Jie Zhang, Wentong Ge, Yongli Guo, Yongbo Yu, Guoli Wang, Tianlu Mei, Qiaoyin Liu, Nian Sun, Yuzhu He, Xiaodan Li, Yuwei Liu, Jun Tai, Xin Ni

**Affiliations:** ^1^Department of Otolaryngology, Head and Neck Surgery, National Center for Children's Health (NCCH), Beijing Children's Hospital, Capital Medical University, Beijing, China; ^2^Beijing Key Laboratory for Pediatric Diseases of Otolaryngology, Head and Neck Surgery, National Center for Children's Health (NCCH), Beijing Pediatric Research Institute, Beijing Children's Hospital, Capital Medical University, Beijing, China; ^3^Department of Gastroenterology, National Center for Children's Health (NCCH), Beijing Children's Hospital, Capital Medical University, Beijing, China; ^4^Department of Otorhinolaryngology, Children's Hospital, Capital Institute of Pediatrics, Beijing, China

**Keywords:** congenital pyriform sinus fistula, gastroscopy, diagnostic value, receiver operating characteristic curve, suspension laryngoscopy

## Abstract

**Objective:** The aim of this study was to explore the diagnostic value of gastroscopy under local anesthesia for congenital pyriform sinus fistula (CPSF).

**Methods:** This research was a diagnostic study. Patients received gastroscopy under local anesthesia to diagnose CPSF, and suspension laryngoscopy under general anesthesia was performed 2 days after gastroscopy. Various conditions of the internal opening of CPSF were then recorded. Patients were grouped according to lesion sides, age, time after the inflammation subsided, and history of previous surgery. The sensitivity, specificity, area under the receiver operating characteristic curve (AUC), accuracy, and positive and negative predictive values of gastroscopy were compared between the groups.

**Results:** A total of 48 patients were recruited in this study, and no patients had severe gastroscopy-related complications. The diagnostic values of gastroscopy in 41 cases (85.4%) were consistent with suspension laryngoscopy. The sensitivity of gastroscopy was 86.4%, the specificity was 75%, the AUC was 0.807, the positive prediction rate was 97.4%, the negative prediction rate was 33.3%, the accuracy rate was 85.4%, and the diagnostic odds ratio (DOR) was 2.1. The kappa consistency test results had statistical significance (*P* = 0.0026, kappa = 0.3913). The diagnostic value of gastroscopy was better for the patients with inflammation subsiding for more than 4 weeks (*P* < 0.0001).

**Conclusion:** Gastroscopy under local anesthesia is a safe, effective, reliable and novel diagnostic method for CPSF, and it is especially recommended as a diagnostic method for the patients with inflammation subsiding for more than 4 weeks.

## Introduction

Congenital pyriform sinus fistula (CPSF) is a rare neck deformity of congenital branchial origin, which is caused by the abnormal degeneration of the 3rd or 4th branchial cleft in early embryonic development. It has been reported that 80% of cases occurred in children (1, 2). Conventional auxiliary diagnostic methods included clinical manifestations, cervical ultrasound, barium esophagram, cervical computed tomography (CT), or magnetic resonance imaging (MRI). However, currently, these methods, as diagnostic accuracy rates are not particularly high, cannot be used as gold standards. Meanwhile, complicated clinical manifestations of CPSF may increase the misdiagnosis rate and expose patients to risks of delayed treatment and repeated infection. In 2014, Chen et al. (3) demonstrated that suspension laryngoscopy is a good choice for CPSF diagnosis. However, suspension laryngoscopy should be performed under general anesthesia and is unable to be widely used as a routine examination in outpatient department. Thus, the formulation of a precise surgical procedure may be difficult to determine if it is impossible to confirm a clear diagnosis prior to surgery treatment. Therefore, there is an urgent need for a novel convenient method with less side effect and high diagnostic accuracy rate for CPSF diagnosis before surgery. This study aimed to explore the potential usage of gastroscopy, investigate its reliability and safety, and find out clinical factors that could affect its diagnostic efficacy in CPSF diagnosis.

## Materials and Methods

### Ethical Considerations

This study was performed in compliance with the Declaration of Helsinki and was approved by the Ethics Committees of Beijing Children's Hospital, Capital Medical University. Informed consent was signed by patients or legal guardians.

### Inclusion and Exclusion Criteria

The inclusion criteria of this research were as follows: (1) patients younger than 18 years old, (2) children who were suspected of CPSF by ultrasonography or MRI examinations, and (3) patients and/or guardians who provided written informed consent. The exclusion criteria were as follows: (1) patients who were in the acute infection period or shorter than 7 days after the last infection, (2) patients could not tolerate anesthesia, and (3) parents provided incomplete medical history.

### Gastroscopy Exploration Under Local Anesthesia

**Figure 1:**
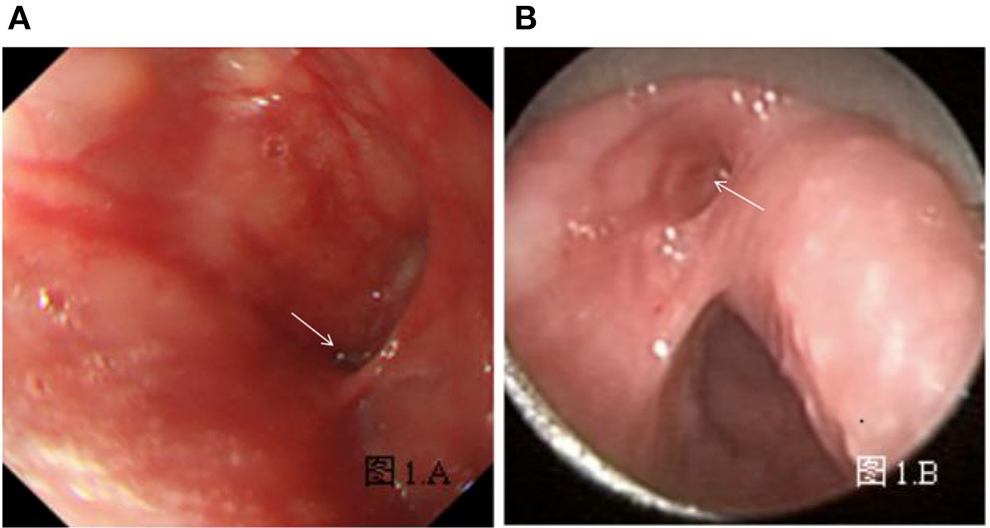
**(A)** Left internal opening of CPSF and Betz folds using gastroscopy under local anesthesia. **(B)** Left internal opening of CPSF, Betz folds, and esophageal entrance using support laryngoscopy under general anesthesia. The arrow indicates the internal opening of CPSF.

All patients were deprived of food and water (≧6 h) and did not receive sedative measures before gastroscopy. Local anesthesia was conducted with 10 ml oral lidocaine gel 20 min prior to examination. An electronic fiberoptic endoscope with a diameter of 0.52 or 0.92 cm was selected according to the children's age, height, and weight. The left lateral decubitus position was assumed for the examination. After a dental pad was placed, the doctor manipulated the endoscope into the oral cavity until the esophageal entrance. Then, the pyriform sinus and Betz folds were fully exposed. The pyriform sinus on the affected side was first explored; subsequently, repeated ventilation and irrigation with sterile water were utilized (the extension of local folds as the standard) to search for the internal opening of CPSF (Figure 1A). Then, the same strategy was performed in the contralateral pyriform sinus. In addition, the examination would be stopped and patients would receive symptomatic treatment immediately if they suffered severe cough, mucosal bleeding, and unstable vital signs. In retrospect of our study, none of the patients we recruited dropped out, and only three had minor bleeding in the mucosa of laryngopharyngeal after the examination intervention. Gastroscopy was performed by one gastroenterologist, and the presence of the internal fistula opening was confirmed by two physicians (one gastroenterologist and one otolaryngologist). Disagreements were resolved by consensus with another experienced physician. In this study, only one case was finally resolved by another experienced physician, in which it was difficult to determine the internal opening as the surrounding adhered tissue blocking the fistula.

### Treatment After Examination

Two days after the gastroscopy, patients underwent surgical treatment under general anesthesia. During the operation, the suspension laryngoscope was placed to expose the pyriform sinus and Betz folds and further search for the internal opening of the fistula (Figure 1B). The result of suspension laryngoscopy, the gold standard of CPSF diagnosis, was used to verify the accuracy of gastroscopy as a new diagnostic method. Then, patients with internal openings underwent CO_2_ laser cauterization (4). However, open surgery procedures through the external cervical were performed for three patients as their internal fistula openings cannot be observed.

### Statistical Methods

Qualitative data including sex, the side of CPSF, and clinical manifestations were described using percentages. Quantitative data including the time after the inflammation subsided were presented as median and interquartile range, whereas the age of the patients was described using mean and standard deviation according to normal distribution. Suspension laryngoscopy under general anesthesia was used as the gold standard. The receiver operating characteristic curve (ROC), consisting of sensitivity, specificity, positive predictive value, negative predictive value, diagnostic odds ratio (DOR), and accuracy, was used to evaluate the diagnostic value of gastroscopy for CPSF. Then, clinical factors that could affect CPSF diagnostic efficacy were discussed through different sides of CPSF, age, time after the inflammation subsided, and history of previous surgery. The area under the ROC curve (AUC) comprehensively represented the diagnostic value of CPSF. The kappa consistency test was performed to assess the consistency of diagnostic methods between the gastroscopy and the suspension laryngoscopy. Furthermore, *Z*-test was used to compare the AUC of different clinical factors. *P* < 0.05 was considered to indicate statistically significant difference. The JMP12.0 and SAS 9.4 software were used for statistical analysis.

## Results

### Demographic and Clinical Characteristics

A total of 48 cases suspected of CPSF were recruited at Otolaryngology and Head and Neck Surgery Department of Beijing Children's Hospital from March 2016 to December 2017. In this study, the baseline of patients was consistent with existing reports (5, 6). The demographics and clinical characteristics of subjects are shown in Table 1.

**Table 1:** Demographic and clinical characteristics.

**Total number of patients**	**48**
**Age (month)**
**Mean (standard deviation)**	**56 (42.5)**
**Sex**
**Male:female (ratio)**	**23:25 (0.92)**
**Side, case (%)**
**Left**	**43 (89.6)**
**Right**	**5 (10.4)**
**Past surgery history, case (%)**
**No (initial treatment)**	**43 (89.6)**
**Removal of masses by an external cervical approach (retreatment)**	**5 (10.4)**
**Clinical manifestations, case (%)**
**Neck mass**	**2 (4.2)**
**Repeated redness and swelling**	**36 (75.0)**
**Infectious pus**	**22 (45.8)**
**Time after the inflammation subsided (from the last infection)**
**Median, week (range)**	**4 (1–32)**

*Definition of the last infection: mass/redness and swelling/rupture and pus at the neck, with or without fever, and elevated white blood cell count and C-reactive protein level with or without a hypoechoic abscess in an imaging examination*.

### Diagnostic Efficacy and Security of Gastroscopy

**Figure 2:**
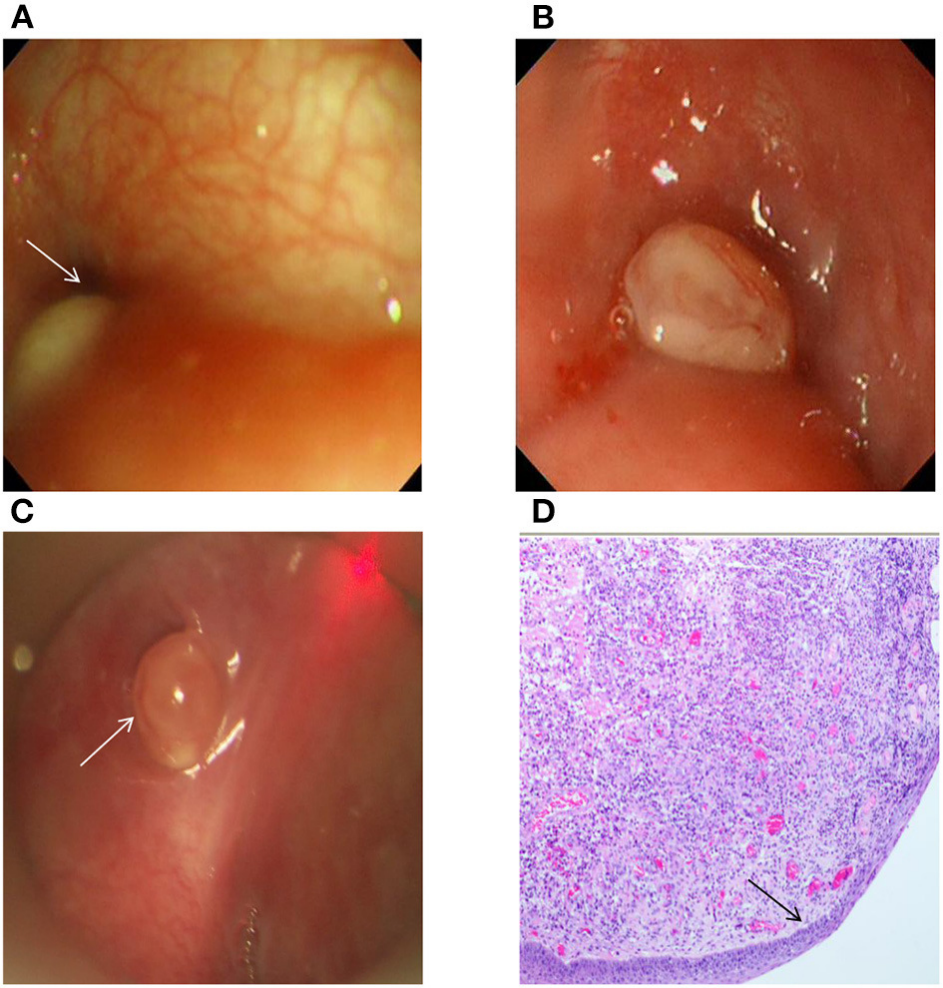
**(A)** The right pyriform sinus under the gastroscope had an oval mass with a size of 0.5 cm × 0.2 cm, the surface was smooth, and the internal fistula opening in the left pyriform sinus could be observed. **(B)** The left pyriform sinus under the gastroscope had an oval mass with a size of ~0.3 cm × 0.4 cm, the surface was smooth and covered by a small amount of mucus, and there was no clear fistula opening. **(C)** Granulation in the left pyriform sinus was observed in the patient in panel **(B)** by support laryngoscopy, and there was a deep internal fistula opening with a size of ~5 mm. **(D)** The postoperative pathology of the patient in panel **(B)** showed that the surface was covered by squamous epithelia, and that there was inflammatory granulation tissue under the epithelia (H&E, ×40). The arrow indicates the internal opening of CPSF **(A,C)** and squamous epithelia **(D)**.

In the process of our study, 38 true-positive cases, containing 1 case of inflammatory granulation tissue surrounding the internal fistula opening (Figure 2A), successfully found the internal fistula openings with gastroscopy and suspension laryngoscopy exploration, respectively. Conversely, one false-positive case observed the internal fistula opening during gastroscopy exploration but not with suspension laryngoscopy exploration. Six false-negative cases observed the internal fistula opening under suspension laryngoscopy exploration but not with gastroscopy exploration. In one of the six cases, the internal fistula opening was covered by inflammatory granulation tissue (pathology diagnosis; Figures 2B–D). Another one of the six cases was not clearly observed due to severe crying and screaming. In addition, three true-negative cases did not detect the internal fistula opening by gastroscopy or suspension laryngoscopy exploration and were further confirmed as branchial cleft cysts in these patients following surgery and pathology result. During gastroscopy under local anesthesia, three patients had minor bleeding in the mucosa of laryngopharynx, which stopped quickly and no treatment was needed. Meanwhile, the internal fistula openings were detected in two of these three patients. No other complications, such as local anesthetic drug allergy, bucking, suffocation, hoarseness, lip and tooth injuries, or cricoarytenoid joint dislocation, were found in all patients with gastroscopy. The examination results of 48 cases are provided in Table 2.

**Table 2:** Exploration results of the internal opening of CPSF by gastroscopy under local anesthesia and support laryngoscopy under general anesthesia.

**No**.	**Internal fistula discovered under gastroscopy**	**Internal fistula discovered under support laryngoscopy**	**Time after inflammation cure/W**	**Remarks**
1	N	Y	4	
2	Y	Y	4	
3	Y	Y	2	
4	Y	N	2	Cystic branchial cleft deformity
5	Y	Y	1	
6	N	Y	4	
7	Y	Y	2	
8	Y	Y	4	
9	N	Y	6	
10	Y	Y	20	
11	Y	Y	6	
12	Y	Y	4	
13	Y	Y	8	
14	Y	Y	6	
15	Y	Y	12	
16	Y	Y	12	
17	Y	Y	4	
18	Y	Y	8	
19	Y	Y	8	
20	Y	Y	4	
21	Y	Y	10	
22	Y	Y	2	
23	Y	Y	8	
24	Y	Y	4	
25	N	Y	1	
26	N	N	6	Cystic branchial cleft deformity
27	Y	Y	6	
28	N	Y	6	
29	Y	Y	4	
30	Y	Y	4	
31	Y	Y	4	
32	Y	Y	32	
33	Y	Y	8	
34	Y	Y	24	
35	Y	Y	8	
36	N	N	–	Cystic branchial cleft deformity
37	N	N	4	Cystic branchial cleft deformity
38	N	Y	4	Inflammatory granuloma at fistula
39	Y	Y	12	
40	Y	Y	4	
41	Y	Y	12	
42	Y	Y	6	Inflammatory granuloma at fistula
43	Y	Y	2	
44	Y	Y	4	
45	Y	Y	3	
46	Y	Y	–	
47	Y	Y	8	
48	Y	Y	8	

### Diagnostic Reliability of Gastroscopy

Comparing the diagnostic value of CPSF between gastroscopy and suspension laryngoscopy, the sensitivity was 86.4%, the specificity was 75%, the AUC was 0.807, the positive prediction rate was 97.4%, the negative prediction rate was 33.3%, the accuracy rate was 85.4%, and the DOR was 2.1. The kappa consistency test result shows statistical significance (*P* = 0.0026, kappa = 0.3913; Table 3). In conclusion, the diagnostic method of gastroscopy was effective in distinguishing patients from non-patients, and its diagnostic capability is consistent with the gold standard.

**Table 3:** Four-fold table exploration conditions of the internal fistula openings of CPSF using two diagnostic methods.

**Gastroscopy under local anesthesia**	**Support laryngoscopy under general anesthesia**		
	**With internal fistula openings**	**Without internal fistula openings**	**Total**
With internal fistula openings	38	1	39
Without internal fistula openings	6	3	9
Total	44	4	48

### Diagnostic Reliability of Gastroscopy Under Different Conditions

As shown in Table 4, the diagnostic results of right-side lesion with gastroscopy, including sensitivity, specificity, AUC, and positive and negative predictive values, were better than those of left-side lesion. Patients were classified into two groups according to age 6. The consequence illustrated that the sensitivity and negative predictive value were higher in the ≥6 y group, whereas the specificity and positive predictive value were lower in the ≥6 y group. However, no difference (*P* = 0.152) was observed on the diagnostic value between the ≥6 and <6 y groups. For different times after the inflammation subsided, the sensitivity, specificity, AUC, and positive and negative predictive values in the ≥4 weeks group were significantly higher than those in the <4 weeks group (*P* < 0.0001).

**Table 4:** Diagnostic reliability of gastroscopy under local anesthesia.

		**Sensitivity (%)**	**Specificity (%)**	**Area under the ROC curve**	**Positive predictive value (%)**	**Negative predictive value (%)**	***P-*value**
Gastroscopy diagnosis		86.4	75.0	0.807	97.4	33.3	–
Side	Left	85.0	66.7	0.758	97.1	25.0	0.1529
	Right	100.0	100.0	1.000	100.0	100.0	
Age	<6 y	76.2	100.0	0.885	100.0	25.0	0.5955
	≥6 y	100.0	50.0	0.750	28.6		
Time after the inflammation subsided	<4 w	85.7	0.0	0.571	20.0	0.2512	<0.0001
	≥4 w	86.1	100.0	0.931	100.0		
	<6 w	81.8	50.0	0.651	94.7		
	≥6 w	90.5	100.0	0.952	100.0	33.3	
History of previous surgery	Initial treatment	84.6	75.0	0.798	97.1	33.3	–
	Retreatment	100.0	–	–	100.0	–	

## Discussion

The diagnosis of CPSF is a great challenge due to its rarity and little recognition, which accounts for <1% of brachial anomalies (7). In most of the previous studies, the primary imaging modalities used to identify CPSF include ultrasound, barium esophagram, CT, and MRI. Nevertheless, the clinical application of these methods is limited due to different diagnostic accuracy rates (8). CT poses an ionizing radiation hazard to young children and low positive predictive value (46–49%), although it is not affected by the age or local inflammation environment in patients (9). In clinical practice, ultrasonography is widely used to evaluate most neck disease in pediatric patients due to its convenience and safety. However, the low positive predictive value (7.9%) of ultrasonography may lead to misdiagnosis because of its limited ability to depict a hypopharyngeal lesion (10, 11). MRI is beneficial for the diagnosis of CPSF for its unequivocal identification of peripheral tissue lesions, such as shallower pyriform sinus, inflammatory tissues, an abscess, and a gas pocket. However, longer scanning time and static observation of the fistula opening make it rarely suitable to pediatric CPSF diagnosis. Electronic laryngoscopy does not provide pneumatic functions, and the positive diagnostic rate is only 13.3% (12). A study reported that fistulography, as an important basis for the diagnosis of CPSF, had been used as a preliminary screening method for suspected patients (13). However, its diagnostic positive predictive rate is only 50–88% (5, 6, 14) and also susceptible to the compliance of patients, the size of the internal fistula opening, the diameter of the fistula and sinus, and the local inflammation environment. Additionally, sometimes misdiagnosis is inevitable while stimulation of chronic inflammatory and growth of tissues leads to the complex anatomical structures of the neck more difficult to recognize (2). In conclusion, the above diagnostic methods cannot be used as the gold standard for the diagnosis of CPSF, although certain diagnostic values are proven under different conditions.

In 2014, Chen et al. (3) pointed out that the application of suspension laryngoscopy to observe the internal opening of the pyriform sinus was the gold standard for CPSF diagnosis. However, some reports have suggested that suspension laryngoscopy, which must be performed under general anesthesia and a substantial force is applied to the tongue, may lead to some damage, such as lips, tooth, throat, and mucosa of laryngopharynx injury, with rates in the literature between 37.5 and 75% (15). Meanwhile, the complications of general anesthesia, such as arrhythmias, blood pressure changes, and laryngospasm (16), and adverse events about post-operative lingual nerve injury presentations caused by extended intraoperative compression should not be neglected either (17). Therefore, it is of vital importance to explore a novel convenient method with less side effect and high diagnostic value for CPSF definitive diagnosis before general anesthesia at present. The electronic gastroscopy has been extensively used for the diagnosis and treatment of pharyngeal diseases (18). The advantages of gastroscopy include soft and flexible endoscopic body and clear and broad exploration field. Most of all, it has a typical inflatable function to support the soft tissue around the internal fistula (19), which can make the detection of the internal fistula easier and is expected to replace the suspension laryngoscopy. Reports of gastroscope-assisted fistulectomy further suggested the possibility of exploring the internal opening of CPSF by gastroscopy (20). A recent study of CPSF in children demonstrated that the success rate in diagnosing CPSF using gastroscope was 97.0% (21). However, the pediatric gastroscope was only utilized during the definitive operation, and no preoperative endoscopy was performed. Therefore, the preoperative diagnostic value of gastroscopy is still unclear. As a result of this, the successful implementation of our study will provide a basis for gastroscopy to become a minimally invasive and convenient diagnostic method of CPSF. The diagnostic positive predictive rate for gastroscopy in this study was 97.4%, which was significantly higher than those of 82–90% for electronic laryngoscopy, 50–88% for barium swallowing radiography, 63–84% for MRI, 46–49% for CT, and 7.9% for ultrasound. In addition, the sensitivity and accuracy values of gastroscopy were 86.4 and 85.4%, respectively, indicating that gastroscopy was effective, accurate, and reliable in CPSF diagnosis. However, the detection rate of the internal opening of CPSF using gastroscopy under local anesthesia also depends on the proficiency of gastroscopy physicians, so in this study, all gastroscopy procedures were conducted by a senior physician (deputy chief physician) to minimize operational errors. As shown in our result, exploration results were statistically significant (*P* = 0.0026, the kappa consistency test) between gastroscopy under local anesthesia and suspension laryngoscopy under general anesthesia, whereas the coefficient of association is 0.39. To sum up, the diagnostic value indicated that gastroscopy was superior to previous diagnostic methods and was consistent with suspension laryngoscopy.

A previous clinical guideline made clear that gastroscopy can be performed with the help of an assistant without sedation (22). In China, the consensus of experts in digestive endoscopic diagnosis and treatment advocates performing gastroscopy under moderate to deep sedation (23), which was also confirmed by a systematic review (24). In this study, gastroscopy was performed under local laryngopharyngeal anesthesia, since the depth to be examined was limited to the level of the pyriform sinus, instead of the esophagus and stomach. Gastroscopy of the internal openings of CPSF may cause laryngopharyngeal stimulations sometimes, such as nausea and vomiting in patients, although the appropriate amount of local anesthetic drug was applied. In severe cases, minor bleeding was caused by friction between the endoscopic body and the mucous membrane (25). Therefore, standardized and skilled operation can relatively shorten the detection time (<3 min in our operation) and reduce complications. We recommend that digestive endoscopy physicians and otolaryngologists should collaborate in the entire process.

This study showed that the diagnostic value of gastroscopy was higher on the right CPSF lesions than on the left, but the difference was not statistically significant. One possible reason for this difference is that most of the children in this study were examined in the left lateral position due to the usual practice of our endoscopy physician. We speculate that the left lateral position might not be conducive to the extension of the mucosal folds of the left pyriform sinus and then unfavorable to search the internal opening of the left pyriform sinus fistula. For the second reason, 89.6% of the lesions were on the left side in this study, which is consistent with previous epidemiological results (90%) (2, 26). Therefore, high detection efficiency was easy to be obtained in small samples of right lesions. Nevertheless, the sensitivity and specificity of gastroscopy in the diagnostic value of left-side lesions can reach to 85 and 66.7%, respectively. We divided the subjects into two groups depending on the age for further analysis, as children's compliance may affect the diagnostic results. Our consequence illustrated that the diagnostic accuracy (AUC) was slightly higher in the ≧6 y group, but there was no statistical difference between the two groups (*P* = 0.59). Thus, gastroscopy diagnosis may not be influenced by the patient's age. Furthermore, a history of previous surgery was also one of the possible factors affecting the diagnosis by gastroscopy (27). The sensitivity and positive predictive values of CPSF diagnosis in our study were all 100% in the retreatment group, whereas those of the initial treatment group were 84.6 and 97.1%, respectively. However, the specificity and AUC values could not be calculated and compared between these two groups due to only five cases in the retreatment group. In brief, the influence of patient's position and sample size on the diagnostic value of gastroscopy will be one of the directions we will discuss in the future.

The laryngopharyngeal submucosal layer of children is looser, which facilitates congestion, swelling, and exudation during inflammation (28). Both mucosal reactive edema and granulation hyperplasia are important factors to cause obstructions of the internal fistula opening (14). Nicollas et al. (29) recommend that barium esophagram should be performed at 6–8 weeks after the acute inflammation phase. In the present study, we grouped time after the inflammation subsided by 6 weeks, and the results showed that they were not significantly different in two groups (*P* = 0.25). For searching the better cut-off value of time, next, we divided subjects into two groups by 4 weeks (30), and the results showed that the diagnostic value of gastroscopy was higher in the ≥4 weeks group (*P* < 0.0001). Therefore, we recommend that gastroscopy should be performed 4 weeks after acute inflammation subsides. This time point is earlier than the recommended in previous reports, but was beneficial for patients who had repeated infections to obtain earlier diagnosis and surgical treatment.

To our knowledge, this is the first study to explore the diagnostic value for CPSF by gastroscopy. The current study confirmed the effectiveness of gastroscopy in the diagnosis of CPSF. Meanwhile, the accuracy of the internal fistula by gastroscopy also provides scientific basis for the treatment of CPSF by CO_2_ laser cauterization with optical fiber through gastroscope forceps. However, our study also had some limitations. Although the diagnostic value of gastroscopy has been confirmed, a sufficient sample size is needed to verify this method. Moreover, randomized controlled trial (RCT) is our next research theme to analyze the diagnostic value of gastroscopy more objectively.

## Conclusion

In the present study, we concluded gastroscopy under local anesthesia as a novel diagnostic method to diagnose CPSF, as it is safe, effective, and reliable. The diagnostic value of gastroscopy was better for the patients with inflammation subsiding for more than 4 weeks. Moreover, there was no significant difference in ages, lesion sides, and initial treatment or retreatment. Thus, the gastroscopy under local anesthesia is especially recommended as a diagnostic method for the patients of CPSF with inflammation subsiding for more than 4 weeks.

## Data Availability Statement

All datasets generated for this study are included in the article/supplementary material.

## Ethics Statement

This study was performed in compliance with the Declaration of Helsinki and was approved by the Ethics Committees of Beijing Children's Hospital, Capital Medical University. Informed consent to participate in the study was signed by patients or their legal guardians.

## Author Contributions

XN and JT contributed to the conception and design of the study. SW, LM, YanL, XZ, JZ, WG, YG, YY, GW, TM, QL, NS, YH, XL, and YuwL contributed to the acquisition, analysis, and interpretation of the data. SW and LM wrote the manuscript. YanL, XZ, JZ, WG, YG, YY, GW, TM, QL, NS, YH, XL, and YuwL revised the manuscript. All authors read and approved the final manuscript.

## Conflict of Interest

The authors declare that the research was conducted in the absence of any commercial or financial relationships that could be construed as a potential conflict of interest.
